# Higher exposure to childhood adversity associates with lower adult flourishing

**DOI:** 10.1186/s12889-022-13063-6

**Published:** 2022-03-29

**Authors:** Lin Wang, Zhiyuan Yu, Wenyi Chen, Juan Zhang, Amie F. Bettencourt

**Affiliations:** 1 Shanghai Jiao Tong University School of Nursing, Shanghai, China; 2 Johns Hopkins School of Nursing, 525N Wolfe Street, Baltimore, MD 21205 USA; 3 Departments of Nursing, School of Medicine, Shanghai Jiao Tong University, International Peace Maternity and Children Hospital of China Welfare Institution, Shanghai, China; 4 Department of Psychiatry & Behavioral Sciences, Johns Hopkins School of Medicine, Baltimore, USA

**Keywords:** Adverse childhood experiences, Flourishing, Young adults, Mental health, Physical health, Wellbeing

## Abstract

**Background:**

Adverse childhood experiences (ACEs) are prevalent and associated with negative health and social outcomes. However, our understanding of how patterns of ACEs exposure relate to positive outcomes in adulthood remains limited. This study aims to identify patterns of ACEs and examine associations with flourishing in a sample of Chinese young adults.

**Methods:**

This cross-sectional study was conducted from August to November 2020. Young adults, ages 18–35, enrolled in undergraduate or graduate programs at universities in Mainland China were recruited through convenience and snowball sampling to participate in a survey. The exposure to ACEs was measured by the twelve-item Chinese version of the ACE-International Questionnaire. Additional measures included six domains of flourishing assessed using the Chinese version of the Flourishing Measure, and demographic covariates (i.e., gender, age, year in university, marital status). Descriptive statistical analysis and latent class analysis (LCA) were performed using SPSS 27 and Mplus 8.5.

**Results:**

Participants included 9468 young adults (mean age = 20.1 years). Majority of participants were female (75.3%), undergraduate students (96.4%), and single (79.8%). Approximately 56% of participants reported at least one ACE; 7.0% reported four or more ACEs. Emotional neglect (33.2%), household violence (20.6%), and parental separation/divorce (13.9%) were the most frequently reported ACEs. LCA identified three ACEs classes: multiple maltreatment and household violence (4.7%), emotional neglect and household violence (16.2%), and low ACEs (79.1%). Individuals in the low ACEs class had the highest level of flourishing whereas individuals in multiple maltreatment and household violence had the lowest level of flourishing in all six domains. There were no significant differences in flourishing between the multiple maltreatment and household violence and the emotional neglect and household violence classes except in the physical and mental health (means = 6.17 vs 6.51, *p* = 0.02) and the financial and material stability domains (means = 5.25 vs 5.66, *p* = 0.04).

**Conclusions:**

Patterns of multiple ACEs exposures were associated with lower levels of flourishing. Our findings have implications for efforts to prevent ACEs exposure through monitoring and promoting family well-being and routine screening to identify those with ACEs exposure to prevent negative social and health sequelae.

**Supplementary Information:**

The online version contains supplementary material available at 10.1186/s12889-022-13063-6.

## Background

Adverse childhood experiences (ACEs) are potentially traumatic events, such as child maltreatment, and residing in stressful environments (e.g., domestic violence, community violence, or living with household members with mental illness), experienced before age 18 [1]. More than half of the population worldwide has reported exposure to at least one ACE, and about one in seven have reported exposure to four or more ACEs [1]. The World Health Organization (WHO) defines health as “a state of complete physical, mental and social well-being and not merely the absence of disease or infirmity” [2]. Although ample research reveals strong associations between ACEs and many specific negative health and social outcomes, including nine of the ten leading causes of death [3–7], this deficit-based approach fails to capture the impact of ACEs on flourishing or overall well-being.

### ACEs and flourishing

Flourishing is an emerging, multidimensional concept of overall well-being and optimal human functioning [8, 9]. It is defined as “living a personally meaningful and engaged life” and explained “somewhat by positive emotion, but mostly by good psychological and social functioning” [10, 11]. Flourishing encompasses the central components of well-being, including not only physical and mental health, but also happiness and life satisfaction, meaning and purpose, character and virtue, and close social relationships [8]. Therefore, flourishing is not merely a reversal of negative aspect of health. Instead, flourishing is distinct from and exists amidst illnesses and adversities and it should be measured as an important outcome in public health [11, 12]. It has been associated with elevated job satisfaction [13], reduced occupational stress [14], reduced proinflammatory gene expression [15, 16], and reduced risk for mortality, cardiovascular events, and developing future mental illnesses in adulthood [12, 17]. Existing literature has documented several predictors of flourishing, including high baseline mental health, social support and social capital, mindfulness, and psychological flexibility [10, 12, 18]. However, the associations between flourishing and ACEs are not well understood. Understanding the associations between ACEs and flourishing in adulthood is significant for public health because promoting flourishing is a more holistic approach to disease prevention and health promotion beyond morbidity and mortality and specifying the association between ACEs and flourishing in adults may illuminate early influences on flourishing. Initial empirical evidence from a US adult sample suggests negative associations between ACEs and flourishing [19]. However, the relationship between ACEs and adult flourishing in non-Western contexts (e.g., Mainland China) is not well understood.

### ACEs and flourishing among the Chinese population

ACEs are prevalent in China, with 50 to 74% of the Chinese population reporting exposure to at least one ACE [19–23]. Similar to evidence from Western contexts, exposure to ACEs is significantly associated with negative health outcomes such as risky health behaviors, chronic illnesses, depression, suicide intentions, and posttraumatic stress disorder (PTSD) in Chinese populations [20, 23–25]. Given that flourishing is an emerging concept, little is known about flourishing in the Chinese population. Understanding adult flourishing in relation to ACEs may inform health promotion strategies among the Chinese population, particularly those exposed to high rates of ACE.

### Patterns of exposure to ACEs

ACEs tend to co-occur [26] and researchers often operationalize ACEs using a cumulative risk approach [24, 27]. Although this approach supports our understanding of the cumulative impact of exposure to ACEs, it fails to identify distinct patterns of exposure that may differentially impact health outcomes. In addition, the cumulative risk approach assumes equal weighting of each adversity, while in reality, each ACE may exert a different impact on health outcomes [28]. Latent class analysis (LCA) allows a data-driven, person-centered approach to operationalizing ACEs, which accounts for the clustering of adversities and identifies potential patterns of ACEs [25, 29, 30]. Recent studies demonstrate that different patterns of ACEs exist, and certain patterns of ACEs (e.g., “Polyadversity” and “Maltreatment and conflict”) have stronger associations with poor health outcomes (e.g., chronic inflammation, anxiety symptoms, and PTSD) than others [29, 31]. Nevertheless, how distinct patterns of ACEs are associated with various domains of flourishing among Chinese young adults remains unknown. Therefore, the purposes of this study are to determine the prevalence and patterns of ACEs and examine the association between patterns of ACEs and flourishing in a community sample of Chinese young adults.

## Methods

This study used a cross-sectional, descriptive design and was conducted from August to November 2020 in Mainland China. The first author’s university ethics review board approved this study.

### Sample and settings

Participants were recruited through convenience and snowball sampling. Young adults eligible for this study were 18 to 35 years old, as defined by the Erikson’s theory of psychosocial development [32], and had enrolled in an undergraduate or graduate program at universities in Mainland China. Participants were recruited virtually. An invitation to the study survey site link was distributed via student cohorts’ online groups on WeChat, the most used communication software in Mainland China.

### Key measures

#### Adverse childhood experiences (ACEs)

ACEs were measured using the Chinese version of the ACE-International Questionnaire (C-ACE-IQ; [33]). The C-ACE-IQ, adapted from the WHO ACE-International Questionnaire [34], includes 12 categories of childhood adversities: physical abuse, emotional abuse, sexual abuse, family substance abuse, incarcerated household member, family mental illness, household violence, parental separation or divorce, emotional and physical neglect, bullying, and community violence. The C-ACE-IQ demonstrated good content validity (scale content validity index (S-CVI) = 0.89), and test-retest reliability (intraclass correlation = 0.88) in a sample of Chinese university students (*n* = 566) [33].

The ACE-IQ can be scored using two methods recommended by the WHO [34], the binary and frequency methods. Both methods dichotomize the 12 ACEs categories into “non-exposure” and “exposure,” resulting in a total score range from 0 to 12. However, the frequency version requires a higher threshold for a positive ACEs exposure. For example, the household violence response options include “many times,” “a few times,” “once,” and “never.” A positive exposure to household violence using the frequency method requires the endorsement of “many times” or “a few times.” While a positive exposure to household violence using the binary method only requires the endorsement of at least “once.” This study used the frequency method to generate a conservative estimate of ACEs exposure and facilitate cross-population comparisons [6].

#### Flourishing

Flourishing was assessed using the Chinese version of the Flourishing Measure [8]. The Flourishing Measure operationalizes the WHO’s definition of health and contains six domains: (1) happiness and life satisfaction, (2) mental and physical health, (3) meaning and purpose, (4) character and virtue, (5) close social relationships, and (6) financial and material stability. Each domain contains two Likert scale questions, with each question’s scores ranging from 0 to 10 (e.g., 0 = Extremely disagree and 10 = Extremely agree). There are two summary flourishing scores. The “Flourish Index (FI)” is the average of scores from each of the first five domains which indicates flourishing at a given time [8]. The “Secure Flourish Index (SFI)” is the average of scores from all six domains which indicates flourishing over an extended period of time [8]. Both indices range from 0 to 10, with higher scores indicating respondents perceive themselves more positively in terms of human flourishing. The Chinese version of FI and SFI have shown good internal consistency (Cronbach’s α = 0.88 and 0.81, respectively) in a previous study with Chinese clothing supply chain workers [35]. The internal consistency of FI and SFI in this study sample was 0.91 and 0.89, respectively.

#### Other covariates

Demographic characteristics including gender (female vs. male), age (18–35 years), year in university (freshman, sophomore, junior, senior, and graduate school), and marital status (single, married or cohabitating, divorced, separated, widowed, and other) were also collected.

### Data collection

Interested students entered the study through the survey link. A short description of the study purpose and survey content was presented on the first page of the online survey. Implied consent to participate was indicated when participants responded to survey items. The survey was anonymous and programmed to allow single completion per device to prevent duplicate submissions. From August to November 2020, a total of 11,305 individuals responded to the survey. While 676 individuals were excluded due to ineligibility (e.g., less than 18 years old), 1161 were excluded due to missing over 25% of survey question responses, leaving a final sample of 9468 responses included in the data analysis. See Supplemental eTable1 for comparisons on the observed characteristics between the included sample (i.e., respondents who completed at least 75% of all survey measures) and those excluded due to missing data.

### Data analysis

Data were analyzed using SPSS 27.0 [36] and Mplus 8.5 [37]. Descriptive statistics were used to describe study variables, including means, standard deviations (SDs), and frequencies. Missing data patterns were assessed using the EM procedure and confirmed that data were missing at random. The level of statistical significance was set at alpha = 0.05.

Latent class analysis (LCA), a person-centered approach used to group individuals into unobserved latent classes based on patterns of responses to a set of observed variables [38], was used to examine patterns of ACEs. We compared a series of one to five class solutions on model fit statistics, classification accuracy, and class size to select the final latent class model. Model fit was measured with the Bayesian information criterion (BIC) and the sample size-adjusted BIC (aBIC) [39]. Decreases in the BIC and aBIC indicate improvements in model fit. The Lo-Mendell-Rubin Likelihood Ratio test (LMRT) and the Bootstrap Likelihood Ratio Test (BLRT) were used to compare a model with k classes relative to a model with k-1 classes. A significant LMRT or BLRT indicates that the k-1 class model should be rejected in favor of the k latent class model. Entropy was also examined; higher entropy values indicated better classification accuracy. Finally, relative class sizes were examined as prior research indicates that uncommon or small classes can be difficult to reliably identify [40]. We assigned individuals to the class based on posterior class probabilities [39].

After classes were identified, we examined differences within ACEs classes on mean scores of six domains and two indices of flourishing. These analyses were conducted separately for each latent class using the BCH auxiliary function in Mplus. The benefits of using the BCH approach are that it accounts for classification error and avoids shifts in class size by using a measurement error weighted model to identify differences in the continuous flourishing variables [41, 42]. Differences within ACEs classes on demographic characteristics were examined using ANOVA and Tukey post hoc comparison for age and chi-square test for categorical variable (i.e., gender, year in university, and marital status).

## Results

### Participants characteristics

Table 1 presents participants’ demographic characteristics. The full sample includes 9468 young adults with a mean age of 20.1 years (SD = 1.7). Three-quarters of the participants were female (75.3%), and most were undergraduate students (96.4%) and single (79.8%). Participants’ mean FI and SFI are 6.93 (SD = 1.65) and 6.87 (SD = 1.61), respectively. The means and standard deviations of all flourishing domains and indices are shown in Table 1.

**Table 1:** Participant characteristics and flourishing means (*N* = 9468)

**Age (in years)**	
Range	18–35
Mean (SD)	20.1 (1.7)
**Gender, n (%)**	
Female	7129 (75.3)
Male	2244 (23.7)
Missing	95 (1.0)
**Year in university, n (%)**	
Freshman	2146 (22.7)
Sophomore	2652 (28.0)
Junior	2986 (31.5)
Senior	1342 (14.2)
Graduate	259 (2.7)
Missing	83 (0.9)
**Marital status, n (%)**	
Single	7554 (79.8)
Married or cohabitate	107 (1.1)
Others^a^	1807 (19.1)
**Flourishing Measures, mean (SD)**	
Flourish Index	6.93 (1.65)
Secure Flourish Index	6.87 (1.61)
Domain 1: Happiness and life satisfaction	6.91 (1.96)
Domain 2: Physical and mental health	7.50 (1.80)
Domain 3: Meaning and purpose	6.90 (1.92)
Domain 4: Character and virtue	6.67 (1.89)
Domain 5: Close social relationships	6.70 (1.99)
Domain 6: Financial and material stability	6.55 (2.42)

*Note*. ^a^Other includes missing, divorced, separated, widowed, or other marital status. The “Flourish index” is the average of the first five domains. The “Secure flourish index” is the average of all six domains

### Prevalence of ACEs

Table 2 shows participants’ exposure to ACEs by total scores and categories. Fifty-six percent of participants reported at least one ACE; 7.0% reported four or more ACEs. Total ACEs scores ranged from 0 to 12 (M = 1.09; SD = 1.42). Emotional neglect (33.2%), household violence (20.6%), and parental separation or divorce (13.9%) were the most frequently reported ACEs.

**Table 2:** ACEs exposures by total scores and categories (*N* = 9468)

**ACEs total scores**	**n**	**%**
0 ACE	4151	43.8
1–3 ACEs	4662	49.2
4 or more ACEs	655	7.0
**ACEs categories**	**n**	**%**
Emotional neglect	3145	33.2
Household violence	1952	20.6
Parental separation or divorce	1319	13.9
Sexual abuse	969	10.2
Community violence	771	8.1
Emotional abuse	669	7.1
Physical abuse	410	4.3
Physical neglect	312	3.3
Family mental illness	238	2.5
Family substance abuse	222	2.3
Incarcerated household member	217	2.3
Bullying	128	1.4

*Note*. *ACEs* Adverse childhood experiences

### Patterns of ACEs

**Fig. 1:**
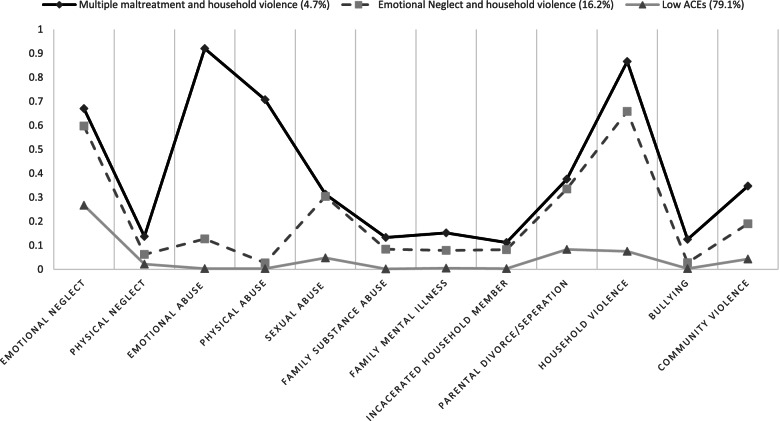
Class item-response probabilities for 12-category ACEs

The three-class model was selected based on the BIC, aBIC, entropy, LMRT (*p* < 0.001), BLRT (*p*<0.001), and class sample size. The LCA model fit statistics are presented in Table 3. Even though the four-class model had the lowest BIC and aBIC, an examination of a scree plot of BIC and aBIC suggested the values leveled off after the three-class. In addition, the four-class model included a very small (0.5%) class which was not particularly meaningful. The item-response probabilities of each ACE for the three classes are presented in Fig. 1. We labeled the three classes as Multiple maltreatment and household violence (4.7%, *n* = 443), Emotional neglect and household violence (16.2%, *n* = 1535), and Low ACEs (79.1%, *n* = 7490).

**Table 3:** Goodness-of-fit statistics and likelihood ratio tests of latent class analysis models containing different numbers of class for ACEs

Latent class model	Number of free parameters	Log likelihood	BIC	aBIC	Entropy	LMRT	BLRT	Smallest class
1	12	−29,167	58,445	58,407	N/A	N/A	N/A	NA
2	25	−26,632	53,493	53,413	0.817	<0.001	<0.001	14.6%
3	38	−26,394	53,136	53,015	0.758	<0.001	<0.001	4.7%
4	51	−26,272	53,010	52,848	0.795	0.003	0.003	0.5%
5	64	−26,232	53,050	52,847	0.813	0.5945	0.596	0.6%

*Note*. *ACEs* Adverse childhood experiences, *BIC* Bayesian information criterion, *aBIC* adjusted Bayesian information criterion, *LMRT* Lo-Mendell-Rubin Likelihood Ratio test, *BLRT* Bootstrap Likelihood Ratio Test

Participants in Multiple maltreatment and household violence had high probabilities of endorsing emotional neglect and abuse, physical abuse, and household violence and relatively low probabilities of endorsing other items. Participants in this class had the highest probabilities of endorsing each of the 12 ACEs compared to participants in other classes. Participants in Emotional neglect and household violence had high probabilities of endorsing emotional neglect and household violence and relatively low probabilities of endorsing other items. The main difference between these classes is that participants in the multiple maltreatment and household violence class had higher probabilities of endorsing emotional and physical abuse compared to participants in the Emotional neglect and household violence class. Participants in the Low ACEs class had the lowest probabilities of endorsing all 12 ACEs compared to the other classes. The most endorsed item for participants in the Low ACEs class was emotional neglect. Across all three classes, participants had low probabilities of endorsing physical neglect, family substance abuse, family mental illness, incarcerated household member, and bullying. Supplemental eTable 2 presents and compares demographic characteristics of individuals in identified latent classes.

### ACEs latent class comparisons on flourishing

**Fig. 2:**
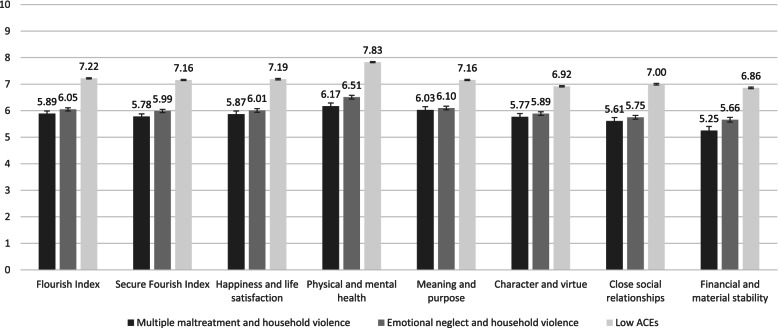
ACEs latent classes on Flourishing indices and domains means

Figure 2 displays means and standard errors on all flourishing indices and domains by identified latent classes. Participants in the Multiple maltreatment and household violence class had the lowest means on all flourishing measures, while those in the Low ACEs class had the highest. Supplemental eTable 3 presents pairwise comparisons. Compared to the Low ACEs class, participants in the Multiple maltreatment and household violence and Emotional neglect and household violence classes reported significantly lower means on all flourishing measures. However, the Multiple maltreatment and household violence and Emotional neglect and household violence classes only differed significantly on the physical and mental health (means = 6.17 vs.6.51, *p* = 0.02) and financial and material stability (means = 5.25 vs.5.66, *p* = 0.04) domains.

## Discussion

Our study examined the prevalence and patterns of ACEs and the association between patterns of ACEs and levels of flourishing in a community sample of Chinese young adults. To our knowledge, this is the first study that examined the relationship between ACEs and adult flourishing in a non-Western context, using Chinese young adults as an example. While prior literature focuses on how ACEs contribute to poor health outcomes, our study extends the science by exploring how ACEs may influence overall well-being in adults including not only physical and mental health, but also happiness and life satisfaction, meaning and purpose, character and virtue, and close social relationships. Findings of our study illuminate the potential impact of early life experiences on adult well-being and have implications for public health efforts on health promotion and disease prevention.

The prevalence of exposure to ACEs found in our study is lower than previous estimates among other Chinese young adult populations (e.g., college students in Hong Kong or rural high school graduates) [43, 44]. However, it is more comparable to estimates from the original ACEs study [45] and general Chinese and US adult populations [7, 20]. Among the 12 categories of ACEs assessed in this study, emotional neglect was the most prevalent, followed by household violence and parental separation/divorce. The prevalence of household violence and parental separation/divorce is comparable to existing studies on ACEs among Chinese populations [20, 23, 25, 43]. While prior studies often identify physical abuse as the most common ACE among Chinese adults, we found emotional neglect was the most common ACE in our sample. This is consistent with a recent study on ACEs among Chinese health science students, which also reported that emotional neglect (31.6%) was the most common ACE [46]. This high rate of emotional neglect is also consistent with the broader literature on child maltreatment among Chinese populations [47, 48]. Despite the high prevalence of emotional neglect, prior research on childhood adversity among Chinese populations typically emphasizes the ACEs’ dimension of threat: experiences that threaten one’s physical integrity, such as abuse and household violence. Our finding calls for greater attention to the ACEs’ dimension of deprivation: the absence of expected environmental inputs such as neglect, which can have significant health and developmental implications such as structural and functional neural development deficits [28].

Our findings also confirm that ACEs often co-occur. The identified three-class pattern aligns with existing studies that have typically identified three or four distinct classes of ACEs in Chinese and East Asian populations [22, 25, 31, 49, 50]. Consistent with prior work, the class characterized by low exposure to all ACEs was the largest class, whereas the class characterized by high exposure to all or multiple ACEs was the smallest class in our study. Although physical neglect is common among Chinese populations [47, 48], young adults in our sample reported low exposure to physical neglect. This sample’s relatively higher socioeconomic status compared to prior work might explain such observations among our participants who completed high school. In addition, the low levels of reported household dysfunction (e.g., family mental illness and substance abuse) across the three classes are similar to prior studies among general Chinese populations [25, 43, 50]. Nevertheless, the low levels of reported household dysfunction should be interpreted with caution. Chinese culture values family honor, and items on household dysfunction such as mental illness, substance abuse, and incarceration carry a significant cultural stigma [51, 52]; thus, participants may underreport exposure to these adversities to “save face” for the family [43].

Among the flourishing domains, our sample reported most positively on physical and mental health and lowest on financial and material stability. Compared to a study that used the same flourishing measure with US employees of two Fortune 500 companies, Chinese young adults in our study scored lower in all flourishing domains and indices except for mental and physical health (Means = 7.41 vs. 7.50) [53]. The lower levels of reported flourishing in our study could be attributed to the major transitions in young adulthood, including social relationships, environment, and financial adjustments, which may affect individuals’ perceptions of their ability to flourish [54, 55]. Whether the differences observed across the two populations are statistically significant and clinically meaningful remain to be explored.

We found that patterns of multiple ACEs exposures were associated with lower levels of flourishing in adulthood Our findings corroborate existing research showing that ACEs have a negative graded-response relationship with flourishing among US children and adults [19, 56, 57]. Our study extends prior research by revealing that the cluster of childhood maltreatment (including emotional neglect) and household violence may exert a substantial impact on one’s ability to flourish well into adulthood. An alternative approach could be used in future studies to examine the differences in the characteristics of groups experiencing the same level of ACEs but with various levels of flourishing to identify strategies to promote individual flourishing.

### Limitation

First, the cross-sectional design limits our ability to infer causal relationships between exposure to ACEs and flourishing. A prospective, longitudinal design should be used in future studies to specify the impact of childhood adversity on well-being in parallel to poor health outcomes. Second, our voluntary sample constitutes relatively well-educated Chinese young adults who are college or graduate students. Although this group’s reported ACEs prevalence rate is comparable to that in the general Chinese and US populations, multiple factors (e.g., geographic area and income) influence the prevalence of ACEs [44, 58]. Thus, our findings may not generalize to other populations. Lastly, since we collected all data through self-report, our study is subject to the shared method and recall biases [59].

## Conclusions

ACEs are common among Chinese young adults, with emotional neglect being the most frequently reported in our sample. Three distinct latent classes of ACEs were identified: low ACEs, emotional neglect and household violence, and multiple maltreatment and household violence. Patterns of multiple ACEs exposures are associated with lower overall well-being. Our findings contribute to a growing literature that emphasizes the role of exposure to trauma and adversities on health and well-being in the long term. Given our findings that ACEs exposure is associated with lower levels of flourishing, it is critical that we focus efforts on the identification and treatment of those who have already experienced ACEs who are at increased risk for negative health outcomes as well as the prevention of ACEs exposure altogether. Thus, there is a need to invest in efforts to promote family well-being through a combination of (1) conducting trauma-informed routine screening of parents and children for ACEs exposure and associated health consequences in the context of primary medical care and connecting those experiencing negative health sequelae with appropriate evidence-based treatments (e.g., Trauma-Focused Cognitive Behavioral Therapy; Child-Parent Psychotherapy) [60, 61]; and (2) implementing preventive interventions directed at proactively strengthening parenting skills, parent-child relationships, and parental resilience in the context of early childhood education and primary care settings [62–64].

## Supplementary Information

**Additional file 1.**

## Data Availability

The datasets used and/or analyzed during the current study are available from the first author on reasonable request. The raw data is not readily available to the public because it contains participants’ confidential information and sensitive data related to their childhood adversity.

## Abbreviations

ACEs: Adverse Childhood Experiences
LCA: Latent Class Analysis
PTSD: Post Traumatic Stress Disorder
C-ACE-IQ: Chinese version of the ACE-International Questionnaire
BIC: Bayesian information criterion
aBIC: Sample size-adjusted BIC
LMRT: Lo-Mendell-Rubin Likelihood Ratio test
BLRT: Bootstrap Likelihood Ratio Test
