# Supplementary material for: Higher exposure to childhood adversity associates with lower adult flourishing
Source: BMC Public Health. 2022 Mar 29;22:612. doi: 10.1186/s12889-022-13063-6 (PMC8966170; doi:10.1186/s12889-022-13063-6)
Supplement: Supplementary file 1 — Additional file 1. [file 12889_2022_13063_MOESM1_ESM.docx]

| **eTable1.**  Comparisons on the respondent characteristics between sample included for analysis and sample excluded due to missing data | | | |
| --- | --- | --- | --- |
| Participant characteristics | Included sample  (N = 9468) | Excluded sample  (n = 1161) | *P*-value for between sample comparison |
| **Age (in years)** | | | |
| Range | 18-35 | 18-35 | 0.438 |
| Mean (SD) | 20.05 (1.67) | 20.09 (1.93) |  |
| **Gender, n (%)** | | | |
| Female | 7129 (75.3) | 686 (59.1) | < 0.001 |
| Male | 2244 (23.7) | 388 (33.4) |  |
| Missing | 95 (1.0) | 87 (7.5) |  |
| **Year in university, n (%)** | | | |
| Freshman | 2146 (22.7) | 239 (20.6) | < 0.001 |
| Sophomore | 2652 (28.0) | 374 (32.2) |  |
| Junior | 2986 (31.5) | 327 (28.2) |  |
| Senior | 1342 (14.2) | 145 (12.5) |  |
| Graduate | 259 (4.0) | 13 (1.1) |  |
| Missing | 83 (0.9) | 63 (5.4) |  |
| **Marital status** | | | |
| Single | 7554 (79.8) | 746 (64.2) | < 0.001 |
| Married or cohabitate | 107 (1.1) | 17 (1.5) |  |
| Other* | 1807 (19.1) | 398 (34.3) |  |
| *Note*. Respondents who completed at least 75% of all survey measures were included in the final sample. Respondents with more than 25% non-response in any survey measures were excluded in the final sample. *Other includes missing, divorced, separated, widowed, or other marital status | | | |

| **eTable 2.**  *Comparison of ACEs latent classes on participant characteristics* | | | | | | | | | | | | | | |  |
| --- | --- | --- | --- | --- | --- | --- | --- | --- | --- | --- | --- | --- | --- | --- | --- |
| Categorical variables | Gender, n (%) | | | Year in university, n (%) | | | | | | | Marital status, n (%) | | | |  |
|  | Female | Male | Missing | | Freshman | Sophomore | Junior | Senior | Graduate | Missing | | Single | Married or cohabitate | Other^1^ | |
|  | χ^2^ (p-value)  22.6** (< 0.001) | | | | χ^2^ (p-value)  45.0** (< 0.001) | | | | | | | χ^2^ (p-value)  15.5* (0.004) | | | |
| Class1: Multiple maltreatment and household violence | 296 (66.8) | 137 (30.9) | 10 (2.3) | | 83 (18.7) | 125 (28.2) | 120 (27.1) | 98 (22.1) | 14 (3.2) | 3 (0.7) | | 344 (77.7) | 9 (2.0) | 90 (20.3) | |
| Class 2: Emotional neglect and household violence | 1170 (76.2) | 353 (23.0) | 12 (0.8) | | 319 (20.8) | 416 (27.1) | 491 (32.0) | 244 (15.9) | 56 (3.6) | 9 (0.6) | | 1190 (77.5) | 27 (1.8) | 318 (20.7) | |
| Class 3: Low ACEs | 5663 (75.6) | 1754 (23.4) | 73 (1.0) | | 1744 (23.3) | 2111 (28.2) | 2375 (21.7) | 1000 (13.4) | 189 (2.5) | 71 (0.9) | | 6020 (80.4) | 71 (0.9) | 1399 (18.7) | |
| Continuous variable | Age, mean (SE) | | | | | | | | | | | | | |  |
| Class 1 | 20.15 (0.079) | | | | | | | | | | | | | |  |
| Class 2 | 20.17 (0.044) | | | | | | | | | | | | | |  |
| Class 3 | 20.02 (0.018) | | | | | | | | | | | | | |  |
|  | Mean difference (p-value) | | | | | | | | | | | | | |  |
| Class 1 vs Class 2 | -0.016 (0.983) | | | | | | | | | | | | | |  |
| Class 1 vs Class 3 | 0.134 (0.211) | | | | | | | | | | | | | |  |
| Class 2 vs Class 3 | 0.149* (0.003) | | | | | | | | | | | | | |  |
| *Note*. ACEs = Adverse childhood experiences. ^1^Other includes missing, divorced, separated, widowed, or other marital status. * p-value < 0.05. ** p-value < 0.001 | | | | | | | | | | | | | | |  |

| **eTable 3**.  *ACEs latent classes on flourishing indices and domains means* | | | | | | | | |
| --- | --- | --- | --- | --- | --- | --- | --- | --- |
|  | Flourish Index | Secure Flourish Index | Domain 1: Happiness and life satisfaction | Domain 2: Physical and mental health | Domain 3: Meaning and purpose | Domain 4: Character and virtue | Domain 5: Close social relationships | Domain 6: Financial and material stability |
| Mean (SE) | | | | | | | | |
| Class 1: Multiple maltreatment and household violence | 5.89 (0.10) | 5.78 (0.10) | 5.87 (0.12) | 6.17 (0.12) | 6.03 (0.12) | 5.77 (0.12) | 5.61 (0.13) | 5.25 (0.16) |
| Class 2: Emotional neglect and household violence | 6.05 (0.06) | 5.99 (0.06) | 6.01 (0.07) | 6.51 (0.07) | 6.10 (0.07) | 5.89 (0.07) | 5.75 (0.07) | 5.66 (0.09) |
| Class 3: Low ACEs | 7.22 (0.02) | 7.16 (0.02) | 7.19 (0.03) | 7.83 (0.02) | 7.16 (0.02) | 6.92 (0.02) | 7.00 (0.03) | 6.86 (0.03) |
|  | χ2 (p-value) | | | | | | | |
| Class 1 vs Class 2 | 1.61 (0.20) | 2.75 (0.10) | 0.86 (0.35) | 5.26 (0.02) | 0.19 (0.66) | 0.67 (0.41) | 0.78 (0.38) | 4.47 (0.04) |
| Class 1 vs Class 3 | 173.65 **(<0.001) | 199.34 **(<0.001) | 121.38 **(<0.001) | 195.22 **(<0.001) | 86.90 **(<0.001) | 91.45 **(<0.001) | 119.77 **(<0.001) | 103.43 **(<0.001) |
| Class 2 vs Class 3 | 307.57 **(<0.001) | 330.61 **(0.001) | 212.31 **(<0.001) | 308.09 **(<0.001) | 174.88 **(<0.001) | 171.84 **(<0.001) | 230.34 **(<0.001) | 134.75 **(<0.001) |
| *Note*: The “Flourish index” is the average of the first five domains. The “Secure flourish index” is the average of all six domains. ** p-value < 0.001 | | | | | | | | |
